# In vitro modeling of solid tumor interactions with perfused blood vessels

**DOI:** 10.1038/s41598-020-77180-1

**Published:** 2020-11-19

**Authors:** Tae Joon Kwak, Esak Lee

**Affiliations:** Nancy E. and Peter C. Meinig School of Biomedical Engineering, Cornell University, Ithaca, NY 14853 USA

**Keywords:** Biological models, Lab-on-a-chip, Environmental biotechnology, Microfluidics, Biological techniques, Biotechnology

## Abstract

Molecular crosstalk between intra-tumor blood vessels and tumor cells plays many critical roles in tumorigenesis and cancer metastasis. However, it has been very difficult to investigate the biochemical mechanisms underlying the overlapping, multifactorial processes that occur at the tumor-vascular interface using conventional murine models alone. Moreover, traditional two-dimensional (2D) culture models used in cancer research do not recapitulate aspects of the 3D tumor microenvironment. In the present study, we introduce a microfluidic model of the solid tumor-vascular interface composed of a human umbilical vein endothelial cell (HUVEC)-lined, perfusable, bioengineered blood vessel and tumor spheroids embedded in an extracellular matrix (ECM). We sought to optimize our model by varying the composition of the tumor spheroids (MDA-MB-231 breast tumor cells + mesenchymal stem cells (MSCs)/human lung fibroblasts (HLFs)/HUVECs) and the extracellular matrix (ECM: collagen, Matrigel, and fibrin gels with or without free HLFs) that we used. Our results indicate that culturing tumor spheroids containing MDA-MB-231 cells + HUVECs in an HLF-laden, fibrin-based ECM within our microfluidic device optimally (1) enhances the sprouting and migration of tumor spheroids, (2) promotes angiogenesis, (3) facilitates vascular invasion, and (4) preserves the structural integrity and functionality of HUVEC-lined microfluidic channels. This model may provide a platform for drug screening and mechanism studies on solid tumor interactions with functional blood vessels.

## Introduction

Intra-tumor blood vessels are known to play critical roles in tumorigenesis and cancer metastasis. The unique morphological and functional properties of these vessels regulate intra-tumor metabolism and promote the anaerobic and glycolytic conditions that characterize the tumor microenvironment. Consequently, many clinically available cancer therapeutics exert their antineoplastic effects by inhibiting tumor angiogenesis, the process by which intra-tumor blood vessels expand to accommodate tumor growth^1^. While the relevance of vascular biology to oncology has thus been clearly established, the precise molecular interactions that occur at the tumor-vascular interface are largely unknown. Increased insight into these processes would allow researchers and clinicians to better understand how the structure of intra-tumor vessels affects the targeted delivery of anti-cancer therapeutics, a tumor’s response to these drugs, and the mechanisms that underlie cancer metastasis^2^. However, a major limitation of current cancer research is that it is very difficult to investigate the biophysical mechanisms underlying multifactorial processes, such as those which occur at the tumor-vascular interface, using conventional murine models. Moreover, although animal models have been used in biomedical research to understand tumor-vessel interactions by us^3–5^ and others^6,7^, it has been difficult to unravel the tumor-vessel interactions, mass transport mechanisms through the vessels, and drug responsiveness in these models due to the natural complexity of live animals. In particular, it has been very challenging to isolate and control the relative contributions of biological and biophysical factors to tumor vessel interactions.

Therefore, there has been a major push in recent years to model tumor-vessel interactions in vitro using biomimetic platforms. In particular, three-dimensional (3D) cultures of tumor cells called tumor spheroids have been widely used^8^. Tumor spheroids are clusters of tumor cells formed due to the tendency of adherent cells to aggregate while growing in media suspension^8^. Multicellular tumor spheroids, which are the conglomeration of several tumor-associated cell types, have been used to model human solid tumors because of their morphological and biological similarities to in vivo human tumors. Indeed, compared to two-dimensional (2D) tumor cell culture models, 3D multicellular tumor spheroids very closely recapitulate the cell–cell and cell-extracellular matrix (ECM) interactions between multiple tumor stromal cells, which are essential characteristics of the heterogeneous tumor microenvironment (TME)^9–12^. Additionally, the size and morphology of tumor spheroids are inherently plastic, making them particularly useful in studying drug-induced changes in the behavior of tumor cells^13^. The 3D tumor spheroids models are thus a relevant model of tumor cell growth, migration, and differentiation^13^.

Recent advances in microfluidics have further advanced the state of spheroid models and have made it possible to co-culture mixed tumor spheroids with realistic, bioengineered blood vessels in vitro^14–17^. Such devices very closely recapitulate the endogenous intra-tumor architecture—in terms of dimensionality, fluid flow (luminal/interstitial), physical deformation, and ECM stiffness—facilitating very detailed analyses into the relationship between blood vessels and tumor spheroids in 3D. The immense utility of these models in cancer research is demonstrated by a recent study, in which the therapeutic effects of anti-cancer drugs were assessed in tumor spheroid models cultured under controlled fluid flow conditions in an engineered tumor vascular network^11^. In this study, tumor cells experiencing fluid flow did not exhibit a dose-dependent response to antineoplastic agents, as has been observed in cells cultured under static flow conditions, allowing researchers to realistically assess the responses of tumor cells to each drug. Additionally, the proliferative behavior of tumor cells in this model more closely recapitulated that of in vivo tumors, creating a physiologically relevant model of tumorigenesis^11^. Another study also demonstrated that the bioengineered blood vessels in such microfluidic models closely mimic the in vivo architecture. Specifically they observed that the differentiation and migration of vascular endothelial cells treated with pro-angiogenic growth factors, as well as the permeability of angiogenic sprouts within the device’s collagen matrix, are comparable to those of endogenous blood vessels^18^. However, despite these exciting developments, it is still challenging to reproduce all aspects of the solid tumor microenvironment (e.g., ECM, stromal cells, growth factors, vascular crosstalk) in microfluidic model systems.

**Figure 1:**
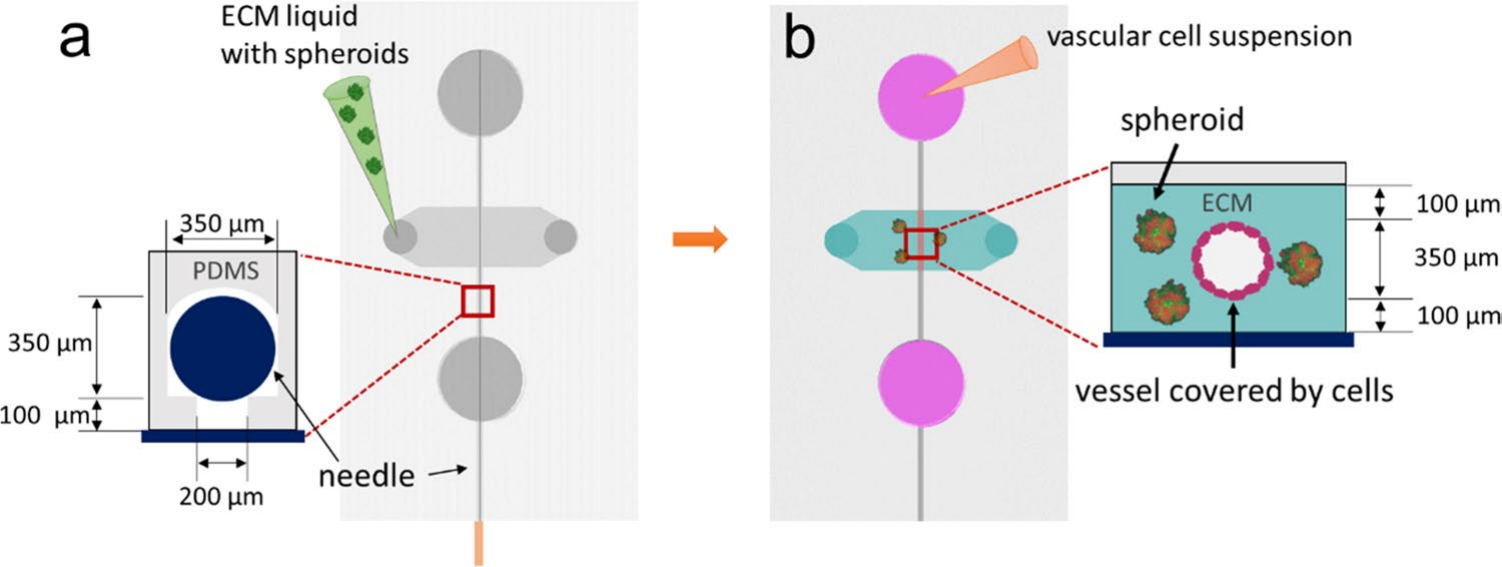
Concept of the device and ECM casting with tumor-spheroids and endothelial cell seeding. (**a**) A casting needle is introduced into the channel in the microfluidic chip device, and the ECM hydrogel liquid is injected with tumor-spheroids and is polymerized as a gel around the needle. (**b**) The casting needle is removed after gelation to create an empty channel embedded in the ECM. Then, HUVECs are introduced into the channel via the media port and allowed to adhere to the ECM channel.

In this study, we worked to optimize the manufacture of microfluidic organ-on-a-chip devices (Fig. 1) to better mimic the physiology of vascularized solid tumors in vitro. Specifically, we aimed to elaborate on the biological determinants of successful interactions between tumor spheroids and perfused blood vessels in our microfluidic system. To do so, we varied the cellular composition of tumor spheroids as well as the ECM environments to which each culture was exposed. We then compared the optimization status of each system by observing (1) the structural integrity of the HUVEC vascular lumen, (2) the sprouting behaviors of tumor spheroids, (3) spheroid-induced angiogenesis, and (4) vascular invasion patterns.

## Results

### Invasion of tumor spheroid mixtures in Matrigel

We first evaluated the growth pattern of spheroids containing MDA-MB-231 metastatic breast cancer cells and mesenchymal stem cells (MSCs), given that MSCs are known to promote tumorigenesis in the tumor microenvironment (TME)^19^. The tumor spheroid mixture groups used in this experiment were: (1) monoculture of MDA-MB-231 cells, (2) a 1:1 ratio co-culture of MDA-MB-231 cells and MSCs, and (3) a 1:3 ratio co-culture of MDA-MB-231 cells and MSCs. Each tumor spheroid was introduced into a microfluidic chip device cast with Matrigel to investigate the interactions between tumor spheroids and bioengineered HUVEC vessels. Each tumor spheroid formed invasive sprouts into the HUVEC blood vessel. Among the three groups, spheroids containing a 1:1 mixture of MDA-MB-231 metastatic breast cancer cells and MSCs formed the largest number of sprouts and the longest invadopodium. Interestingly, the HUVEC vascular channels in each device, regardless of the spheroid culture conditions, collapsed in the Matrigel ECM environment on Day 1. The collapsed vessels did not allow media to flow through the vascular lumen, and thus, we discontinued the investigation at this point (See Supplementary Information Figure S1 for the invasion of tumor spheroid mixtures in Matrigel). These results demonstrate that, when used alone, Matrigel substantially impairs the formation of perusable blood vessels in microfluidic devices.

### Spheroids with MDA-MB-231 and MSCs in Matrigel and collagen I ECM

Given our observation that HUVEC vascular channels collapse in Matrigel ECM, we next explored the behavior of tumor spheroids containing a 1:1 ratio of MDA-MB-231 metastatic breast cancer cells and MSCs which exhibit the most aggressive growth and invasion patterns in several different ECM conditions. We prepared microfluidic chip devices containing these tumor spheroids embedded in 5 different ECM materials: (1) only collagen I, (2) only Matrigel, (3) 1:1 mixture of Matrigel and collagen I (denoted as M-gel and C-gen), (4) 1:2 mixture of Matrigel and collagen I, and (5) 2:1 mixture of Matrigel and collagen I, respectively. In the collagen I dominant ECM microenvironments, such as groups 1 and 4, the HUVEC vasculatures did not collapse, allowing for media perfusion through the vascular lumens. However, the spheroids cultured in such collagen-rich environments (groups 1 and 4) exhibited less aggressive patterns of vascular invasion and sprouting than those cultured in the Matrigel-rich conditions (groups 2 and 5). In fact, no sprouting behavior was observed among spheroids cultured in only collagen I (group 1). By contrast, while Matrigel-dominant ECMs promoted the invasion of growing tumor sprouts, the bioengineered HUVEC vasculatures collapsed under these culture conditions, making them unsuitable for perfusion studies (See Supplementary Information Figure S2 for the spheroids of MDA-MB-231 and MSCs in Matrigel and collagen I matrices).

Consequently, our results demonstrate that none of the ECM conditions tested promoted the invasion/sprouting of tumor spheroids while simultaneously maintaining the luminal integrity of the HUVEC vasculature. Moreover, because we were not able to identify any aggressive angiogenetic activity in spheroid-exposed HUVEC vessels, we discontinued the investigation at this point.

### Tumor spheroid-induced angiogenesis in fibrin ECM

Since neither the collagen I nor Matrigel-based ECM mixtures could (1) preserve the structure of HUVEC vessels, (2) promote angiogenesis, and (3) facilitate the sprouting of tumor spheroids simultaneously, we explored the utility of other culture conditions in modeling the tumor-vascular interface. Specifically, we sought to optimize our model by co-culturing tumor spheroids with human lung fibroblasts (HLFs) in a fibrin gel matrix. HLFs are stromal cells that support vascular sprouting and tumor invasion by secreting growth factors, such as fibroblast growth factor 2 (FGF-2) and vascular endothelial growth factor (VEGF)—both of which promote the formation of healthy blood vessels through angiogenesis^2,20,21^. Fibrin ECM components play a complementary role in tumorigenesis by binding to integrins on the surface of tumor and endothelial cells to facilitate tumor invasion and angiogenesis^2,22^. Therefore, we hypothesized that the synergistic, pro-angiogenic environment created by this culture condition would promote active angiogenesis and tumor sprouting in vitro, while also preserving the luminal structure of our bioengineered tumor blood vessels.

**Figure 2:**
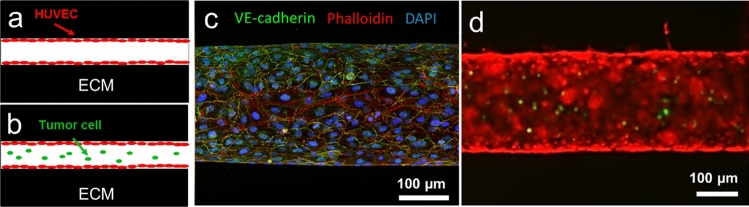
Perfusable engineered blood vessel formation in fibrin ECM. (**a**,**b**) A schematic illustration of the experimental designs for a blood vessel in fibrin ECM (**a**) and functionality test of the lumen structure (**b**). (**c**) Immunostaining of the engineered blood vessels with phalloidin (in red), anti-VE-cadherin antibodies (in green), and DAPI (in blue). (**d**) Functional perfusability test of the lumen structure (in red) with MDA-MB-231 breast cancer cells (in green).

**Figure 3:**
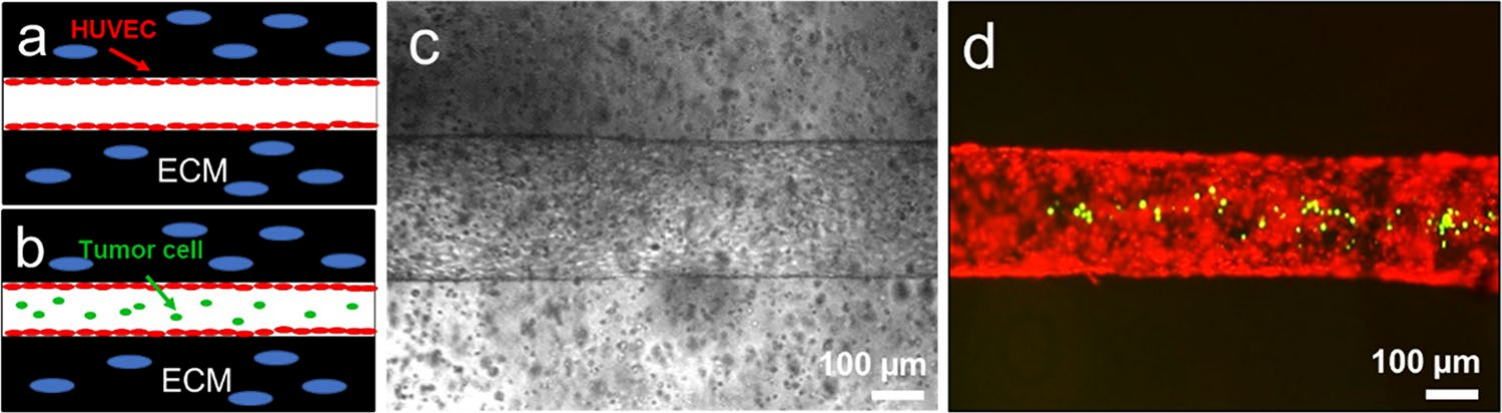
Perfusable engineered blood vessel in HLF-laden fibrin ECM. (**a**,**b**) A schematic illustration of the experimental designs for a blood vessel in HLF-laden fibrin ECM (**a**) and functionality test of the lumen structure (**b**). (**c**) A Bright-field image of the engineered blood vessels with bulk HLF in fibrin ECM. (**d**) Functionality test of the lumen structure (in red) with MDA-MB-231 breast cancer cells (in green) in HLF-laden fibrin ECM.

In this trial, we seeded HUVECs to the fibrin-based ECM in the device to form an engineered blood vessel (Fig. 2a). After seeding the endothelial cells, we introduced fluid shear stress on a rocking platform for 3 days, supplying them shear stress around 4–5 dyne/cm^2^. The engineered blood vessel has been fixed and stained with phalloidin for detecting actin in the cells, anti-VE-cadherin antibodies to visualize endothelial cell–cell adherens junctions, and DAPI to identify the nucleus (Fig. 2c). In addition, the functional perfusability of the lumen structure in fibrin ECM was tested by introducing MDA-MB-231 breast cancer cells (transfected with GFP) into the endothelial lumen (Fig. 2b,d). In addition, we seeded HUVECs to the HLF-laden fibrin ECM (Fig. 3a,c) and tested the functionality as well (Fig. 3b,d).

**Figure 4:**
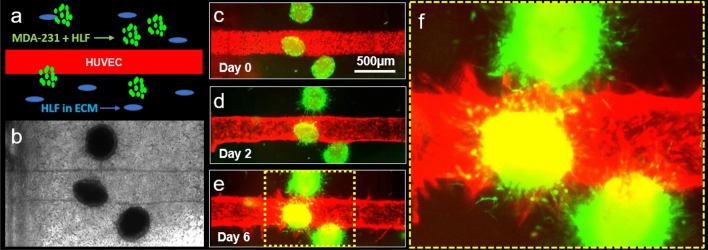
MDA-231 and HLF tumor spheroid-induced angiogenesis. (**a**) A schematic illustration of the experimental design. (**b**) A representative bright-field image of the HUVEC channel and tumor spheroids in the HLF-laden ECM within the microfluidic chip device. (**c**,**d**) Confocal images of MDA-231 and HLF tumor spheroid-induced angiogenesis at day 0 (**c**), day 2 (**d**), and day 6 (**e**). (**f**) An enlarged area of the tumor spheroid-vasculature interface that is highlighted in (**e**).

Subsequently, tumor spheroids composed of MDA-MB-231 cells (denoted as MDA-231) and HLFs were introduced into the fibrin-casted matrix of our microfluidic chip device (Fig. 4). To better mimic the conditions of the human TME, we also introduced HLFs as growth factor suppliers in the ECM bulk and observed the resulting interactions between cultured tumor spheroids and the HUVEC vasculature. Interestingly, we found that this culture condition promoted (1) active sprouting in MDA-231 + HLF tumor spheroids, (2) the formation of a perfusable HUVEC vasculature, and (3) angiogenic sprouting during 6 days of the experiment (Fig. 4c–e). The average number and length of the sprouts formed from the tumor spheroids in this trial were 42 ± 9.42 and 196.08 ± 59.64 µm, respectively. However, while both the tumor spheroids and the HUVEC vasculature actively formed sprouts, they did not invade each other by day 6 of the experiment.

**Figure 5:**
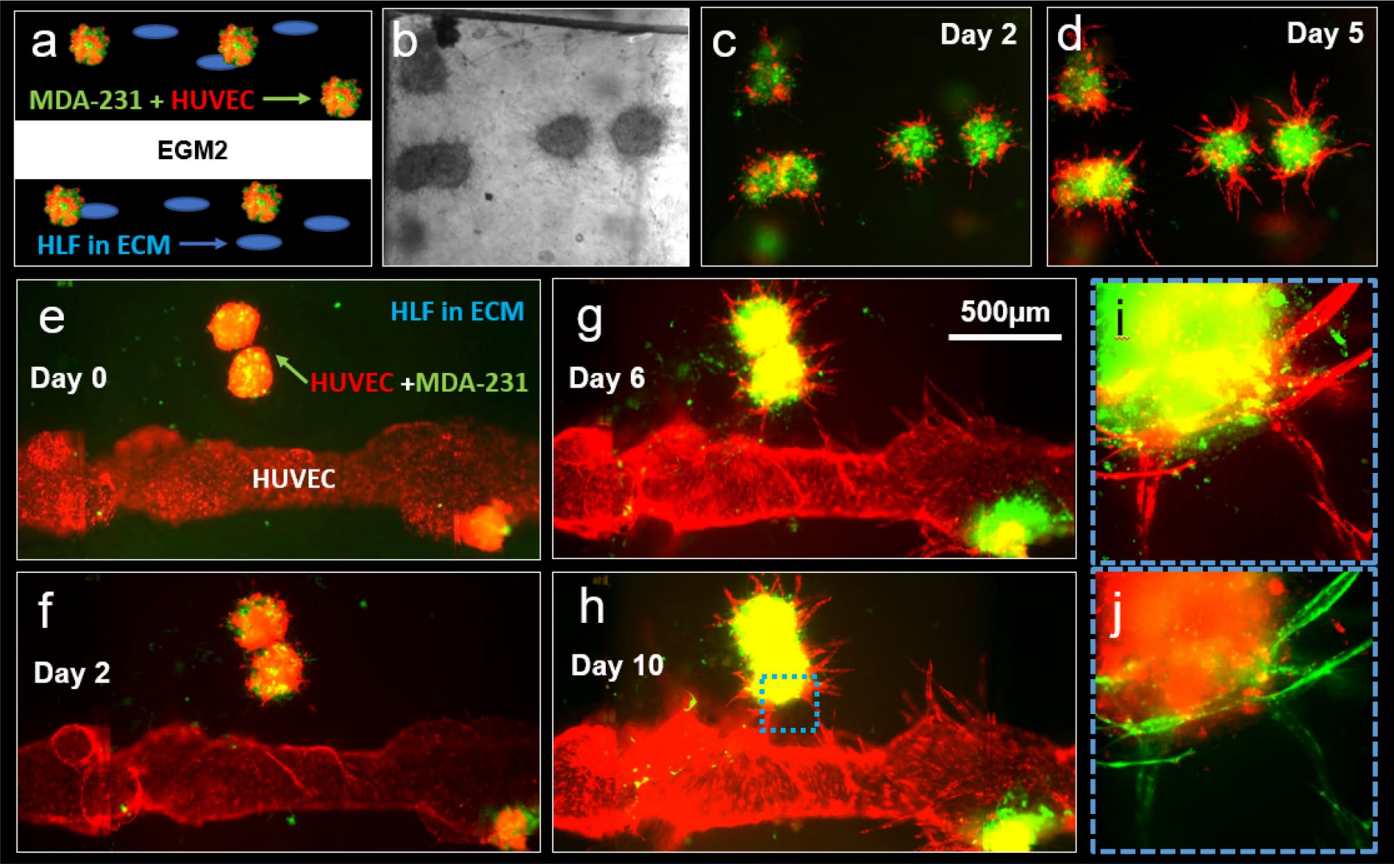
MDA-231 and HUVEC tumor spheroid-induced angiogenesis. (**a**–**d**) Growth of the tumor spheroid mixture consisting of MDA-231 cells and HUVECs. (**a**) A schematic illustration of the experimental design. (**b**) A representative bright-field experimental image of the tumor spheroids and HLF-laden ECM in the microfluidic chip device supplemented with EGM-2. (**c**,**d**) Representative confocal images of the MDA-231 + HUVEC spheroids at day 2 (c) and day 5 (**d**), respectively. (**e**–**h**) Representative confocal images of MDA-231 + HUVEC tumor spheroid-induced angiogenesis at day 0 (**e**), day 2 (**f**), day 6 (**g**), and day 10 (**h**), respectively. (**e**,**f**) Enlarged cross fluorescence confocal images of tumor spheroid-vasculature areas highlighted in (**h**) as original fluorescence (**i**) and reversed pseudo-color (**j**).

In an effort to promote vascular invasion and more closely mimic metastatic conditions, we examined the growth behavior of spheroids composed of MDA-MB-231 cells + HUVECs in an HLF-laden fibrin ECM, as illustrated in Fig. 5a,b. We found that this tumor spheroid mixture exhibited remarkably active proliferation and sprouting behavior for 5 days in culture (Fig. 5c,d). Consequently, we introduced these into our microfluidic chip device alongside a HUVEC-lined channel and examined any tumor-vessel interactions that developed. Intriguingly, this optimized culture condition promoted (1) the active sprouting of MDA-MB-231 cells + HUVECs tumor spheroids, (2) the maintenance of HUVEC vascular lumens, and (3) angiogenesis for a total of 10 days (Fig. 5e–g) The average number and length of sprouts that formed from the tumor spheroids were 26.5 ± 3.5 and 273.59 ± 43.02 µm, respectively. Moreover, sprouts from the tumor spheroids and HUVEC vascular channels successfully invaded one another on day 6 of the experiment (Fig. 5e–h), as confirmed by the enlarged confocal image shown in Fig. 5 as well as the reversed pseudo–colored image shown in Fig. 5j.

**Figure 6:**
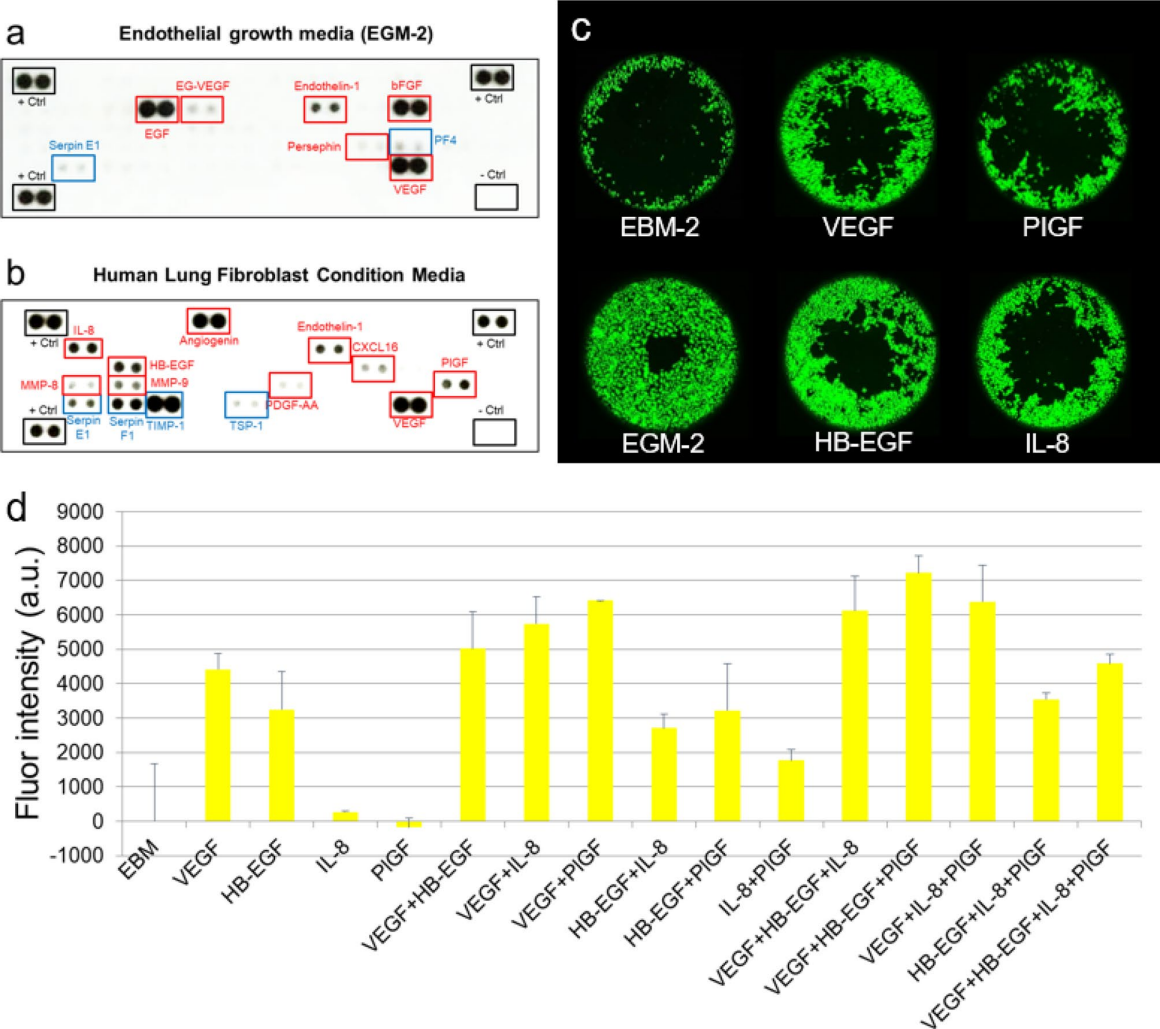
Reverse western blot assays and cell migrations. Reverse western blot assays were performed using human angiogenesis antibody arrays (R&D systems), which detected the relative quantities of 55 cytokines in EGM-2 (**a**) and HLFCM (**b**). Representative images of the cytokine array membranes. Factors in red rectangles represent pro-angiogenic growth factors. Factors in blue rectangles represent anti-angiogenic growth factors. (**c**) The effect of pro-angiogenetic growth factors on HUVECs migration in collagen I coated Platypus migration plates was tested. The migration of HUVECs in EBM-2 (top left) and EGM-2 (bottom left) media as well as EGM-2 media supplemented with VEGF (top middle), PIGF (top right), HB-EGF (bottom middle), and IL-8 (bottom right) pro-angiogenetic growth factors were examined. (**d**) Fluorescence intensity for combinations test of the four pro-angiogenetic growth factors.

Next, we investigated the molecular mechanisms by which HLFs induced angiogenesis and maintained the integrity of HUVEC vascular lumens. To accomplish this, we prepared samples of HLF conditioned media (HLFCM) and identified any angiogenesis-related proteins in the HLF secretome, as described previously^4^. Briefly, when HLFs reached confluence in T175 tissue culture flasks, the normal growth media were replaced with 8 ml of serum-free media. After a 24-h incubation period, the supernatant was centrifuged and filtered through 0.2 mm syringe filters (Corning). The resulting HLFCM was stored in aliquots at -80 °C to avoid multiple freeze–thaw cycles. We then quantified and compared the relative levels of angiogenesis-related proteins in EGM-2, a positive control (Fig. 6a), and HLFCM (Fig. 6b) using membrane-based antibody arrays. To do so, each specimen was introduced onto an array membrane and incubated overnight. The relative levels of 55 cytokines associated with angiogenesis in each sample were then detected. Factors in red rectangles represent pro-angiogenic growth factors, and factors in blue rectangles represent anti-angiogenic growth factors (Fig. 6a,b). As shown in the figures, we found that the pro-angiogenic proteins Endothelin-1 and VEGF as well as the anti-angiogenic protein Serpin E1 (PAI-1) were all upregulated in both EGM-2 and HLFCM samples. Additionally, we observed that the pro-angiogenic proteins EGF, EG-VEGF, Persephin, bFGF as well as the anti-angiogenic protein PF4 were all upregulated in EGM-2, but downregulated in HLFCM. Conversely, we found that pro-angiogenic proteins IL-8, MMP-8, MMP-9, HB-EGF, Angiogenin, PDGF-AA, CXCL16, PIGF as well as the anti-angiogenic proteins Serpin F1 (PDEF) and TIMP-1 were all upregulated in HLFCM, but downregulated in EGM-2.

Lastly, we compared the effects of the pro-angiogenetic growth factors identified in HLFCM on the proliferative and migratory behavior of HUVECs. Specifically, we performed HUVEC migration assays using collagen I-coated Platypus migration kits, the standard method for assessing the angiogenic potential of endothelial cells^23^. The migration of HUVECs in EBM-2 (Fig. 6c, top left) and EGM-2 (Fig. 6c, bottom left) media were tested as controls. We then assessed the migration of HUVECs cultured in EGM-2 media supplemented with 100 ng/ml VEGF (Fig. 6c, top middle), 100 ng/ml PIGF (Fig. 6c, top right), 100 ng/ml HB-EGF (Fig. 6c, bottom middle), and 100 ng/ml IL-8 (Fig. 6c, bottom right) pro-angiogenetic growth factors as following the concentration used in the previous study^24^. Additionally, the effect of different combinations of the four pro-angiogenetic growth factors tested in Fig. 6c on HUVECs were investigated in Fig. 6d. Our results indicated that HUVECs cultured in EGM-2 media supplemented with the above–aforementioned concentrations of VEGF + HB-EGF + PIGF exhibited the most active migratory behavior.

## Discussion

In this study, we optimized the design of microfluidic organ-on-a-chip models of solid breast tumors to more closely recapitulate the proliferative, migratory, angiogenic, and invasive properties of the tumor-vascular interface. In doing so, we found that while Matrigel-based culture conditions facilitated the migration of monoculture (MDA-MB-231 cells) and mixed (MDA-MB-231 cells + MSCs) tumor spheroids in our 3D model, they also significantly disrupted the structural integrity of the microfluidic HUVEC channel (Fig. S1). This is in part because the stiffness of laminin and proteoglycan, the main components of Matrigel, is significantly smaller than that of Collagen I, so when the seeded cells generate contractile force against the walls Matrigel-majored hydrogel might not maintain their original conduit structure^25^. By contrast, while collagen I-based culture conditions promoted the formation of a perfusable HUVEC channel in our model, they also impaired the sprouting and migration of mixed tumor spheroids (Fig. S2). Additionally, neither Matrigel nor collagen I-based culture conditions promoted angiogenesis in HUVEC-lined microfluidic channels exposed to tumor spheroids.

Interestingly, we found that culturing our tumor spheroids in a fibrin ECM preserved the integrity of our model’s HUVEC channels while simultaneously promoting more active patterns of angiogenesis and tumor cell migration than those observed in other ECM cultures (Figs. 4 and 5). Furthermore, the addition of HLFs to the fibrin-based ECM created a culture condition that closely mimicked the tumor microenvironment in which spheroid sprouting, angiogenesis, and vascular invasion could be observed within six days of seeding as similar as described in the previous study^17^. Therefore, we conclude from these data that a microfluidic model in which mixed tumor spheroids containing MDA-MB-231 cells + HUVECs are cultured alongside a bioengineered blood vessel that is cast in an HLF-laden, fibrin-based ECM optimally recapitulates the endogenous structure of the tumor-vascular interface. Based on this finding, we further demonstrate what angiogenic growth factors are secreted from HLF and found the most important factors in our singular and combination treatment in HUVEC migration models. Upon further investigation, we found that HLFs optimize our 3D culture by secreting several pro-angiogenic growth factors, which promote the formation of new vascular sprouts and preserve the structural properties of existing blood vessels (Fig. 6)^4^. Specifically, our reverse western blot array and cell migration data revealed that the combination of VEGF, HB-EGF, and PIGF—all of which are secreted by HLFs—work together to establish a pro-angiogenic cell culture environment, unlike MSCs, which are known as anti-inflammatory cell types secreting numerous anti-angiogenic factors, such as TIMP-1, PEDF, and TSP-1^26–32^. Further research is required to identify the molecular mechanisms by which these four factors cooperatively promote angiogenesis and to develop the next generation of anti-angiogenic cancer therapeutics.

Future cancer researchers can use this optimization protocol to generate bona fide microfluidic models of the tumor-vascular interface. The application of these rigorously validated models will facilitate detailed investigations of the in vivo tumor microenvironment, tumor growth and development, and tumor interactions with adjacent blood vessels and ECM components. Our model still needs further investigation for broader impact and applications. For example, drug testing for anti-angiogenic therapies remain to be performed to evaluate if the model can support the development of more advanced personalized therapeutics for solid tumor cancers.

## Methods

### Cell culture

Human umbilical vein endothelial cells (HUVECs) were purchased from Lonza and cultured in EGM-2 media (Lonza, Switzerland). MDA-MB-231 breast cancer cells were purchased from ATCC and cultured in DMEM (low glucose) + 10% Fetal bovine serum (FBS) + 2 mM L-glutamine + 50 µg/ml Gentamycin. Human mesenchymal stem cells (MSCs, bone marrow-derived) and human lung fibroblasts (HLFs) were purchased from Lonza and cultured in DMEM (low glucose) + 10% FBS + 2 mM L-glutamine + 50 µg/ml Gentamycin. HUVECs were used at passages 3–8, and the stromal cells (MSCs and HLFs) were used at passages 3–6. Green Fluorescent Protein-labelled MDA-MB-231 cells (GFP-MDA-MB-231) were prepared by transducing MDA-MB-231 cells with a lentiviral construct pCSCG-EGFP (Addgene). Similarly, Red Fluorescent-Protein labelled HUVECs (mApple HUVECs) were prepared by transducing HUVECs with a lentiviral construct pBAD-mApple (Addgene). All the cells were maintained in standard tissue culture incubators at 37 °C, 95% humidity, and 5% CO_2_.

### Device fabrication

The polydimethylsiloxane (PDMS) casting mold used in this study was fabricated by following the microfabrication protocol described in our previous study^33^. All PDMS microfluidic chip devices were then fabricated using conventional PDMS casting procedures^34^. Briefly, a 10:1 ratio mixture of PDMS polymer and cross-linker curing agent (SYLGARD 184 Silicone Elastomer Kit, The Dow Chemical Company, MI, USA) was degassed, cast into the PDMS casting mold, and left in an oven for at least two hours at 80 °C. Inlets and outlets were then punched in the polymerized PDMS device to create media reservoirs and ECM ports, and the whole structure was bonded to a glass coverslip with a plasma reacted ion etch (RIE) surface treatment (PE-25 Plasma Cleaner, Plasma Etch Inc., NV, USA). The volume of the ECM cavity in the center of the device is 20 mm^3^, and the distance between the ECM cavity and the media reservoir is 4 mm. The design and dimensions of the microfluidic chip are illustrated in Fig. 1.

### Tumor spheroid preparation

MDA-MB-231 breast cancer cells (ATCC) under normal culture in DMEM were rinsed with phosphate-buffered saline (PBS) twice and detached with trypsin. Trypsinized and detached MDA-MB-231 cells were diluted with excess culture media (DMEM) and centrifuged at 1000$\times g$ for 4 min. The supernatant was removed, and the MDA-MB-231 cell pellet was reconstituted with fresh DMEM to obtain 1.5 $\times$ 10^4^ cells/ml. The cell suspension was transferred into Ultra-Low Attachment (ULA) 96-well round-bottom plates (Sigma). A 200 µl cell suspension of was loaded in each well of the 96-well plate, resulting in 3000 MDA-MB-231 cells per well. For the experiments in which we co-cultured stromal cells with the MDA-MB-231 tumor cells in the spheroids, we fixed the number of MDA-MB-231 tumor cells at 3,000 cells per well, and only the stromal cells were added in different concentrations. For example, stromal cells co-cultured with the tumor cells in the spheroids include (1) MSCs (MDA-MB-231:MSCs = 1:1 or 1:3); (2) HLFs (MDA-MB-231:HLFs = 1:1); and (3) HUVECs (MDA-MB-231:HUVECs = 1:2). The 96-well plates were incubated at 37 °C, 5% CO2, 95% humidity, and spheroid formation was observed daily.

### ECM casting with tumor spheroid

The microfluidic chip device was treated with plasma RIE and immersed in 0.1% Poly-L-Lysine PLL (#0413, ScienCell Research Laboratories, CA, USA) for 4 h and rinsed with DI water three times. Subsequently, 1% glutaraldehyde (G6257, Millipore Sigma, MO, USA) was added to the ECM port and allowed to remain for 15 min. The chip was then rinsed with DI water three times, and was soaked overnight in fresh DI water on an orbital shaker to fully remove any excess glutaraldehyde. The tumor spheroids (e.g., MDA-MB-231 and MSC, MDA-MB-231 and HLF, MDA-MB-231 and HUVEC, and only MDA-MB-231) were cultured and collected from 96-well plates as described above in the Tumor spheroid preparation section. To mold the cylindrical channel in the chip’s ECM, a 350 µm-diameter casting needle was blocked with 1% bovine serum albumin (BSA) and inserted into the needle guide channel. ECM liquid materials, including rat tail collagen type I (354236, Corning, NY, USA), Matrigel (08-774-392, Corning, NY, USA) and fibrinogen (F8630, Millipore Sigma, MO, USA), along with thrombin (Sigma) were then mixed with the tumor spheroids and polymerized in the cavity of the device around the casting needle, as shown in the Fig. 1a. The concentration of 100% Matrigel was 9.8 mg/ml. Thus, 2:1 mixture with collagen gives 6.54 mg/ml, 1:1 mixture gives 4.9 mg/ml, and 1:2 mixture gives 3.27 mg/ml. For some experiments, we also loaded human lung fibroblasts (HLFs) at a density of 500,000 cells/ml with the tumor spheroids in the device cavity to promote vascular sprouting from the vascular channel and tumor spheroids.

### Vascular cell seeding into devices

Once the ECM materials housing tumor spheroids were solidified, the casting needle was removed to form an empty cylindrical channel completely embedded in ECM. Afterward, 1.4 ml of HUVECs were seeded into the empty channel through the device’s media reservoirs (cell seeding density at 1 million cells/ml) and allowed to form a monolayer cylindrical vascular channel as shown in Fig. 1b. To apply physiologically relevant laminar shear stresses (3–4 dyne/cm^2^), the cell-seeded microfluidic chip was placed on a platform rocker that rotates from − 30° through + 30° at 3 rpm. This generates gravity-driven luminal flow through the vascular channel at 37 °C, 5% CO_2_, and 95% humidity. The culture media (EGM-2) in the device was replenished daily.

### Immunoblot assays

Proteome Profiler Antibody Array Kits for human angiogenesis factors (R&D systems, MN, USA) were used to perform reverse western blots. We followed the manufacturer’s operation manual to investigate the relative levels of angiogenesis-related proteins in endothelial growth media-2 (EGM-2) and human lung fibroblast conditioned media (HLFCM).

### Oris cell migration assay

The motility of HUVECs was evaluated using the Oris Pro Cell Migration Assay Kit with collagen I precoated plates (Platypus Technology, Madison, WI). Fifty thousand HUVECs in the control or combined media were added to each well (100 μl/well) of the 96-well plate. The plate contained stoppers to prevent cells from settling in the center area of the wells. Cells were left to adhere for 8 h, then the stoppers were carefully removed. After 18 h, the cells were labeled with CellTracker Green (Invitrogen, Carlsbad, CA), following the manufacturer’s protocol. The cells migrated to the center of the well were analyzed by reading fluorescence at 485/530 nm on a Victor V plate reader (PerkinElmer, Salem, MA) and recorded using a fluorescence microscope. A detection mask was applied to the bottom of the plate in order to detect cells that had migrated to the area that was previously restricted.

### Statistical analysis

Independent sample populations were compared using unpaired, two-sample t-tests with a normal distribution assumption. *P < 0.05 was the threshold for statistical significance. All of the error bars depict standard deviation.

## Supplementary information

Supplementary Information 1.
