# Supplementary material for: In vitro modeling of solid tumor interactions with perfused blood vessels
Source: Sci Rep. 2020 Nov 19;10:20142. doi: 10.1038/s41598-020-77180-1 (PMC7677310; doi:10.1038/s41598-020-77180-1)
Supplement: Supplementary file 1 — Supplementary Information 1. [file 41598_2020_77180_MOESM1_ESM.pdf]

Supplementary Information

In vitro modeling of solid tumor interactions with perfused blood vessels

Tae Joon Kwak[1] and Esak Lee[1,*]

[1] Nancy E. and Peter C. Meinig School of Biomedical Engineering, Cornell University, Ithaca, New York, 14853, United States

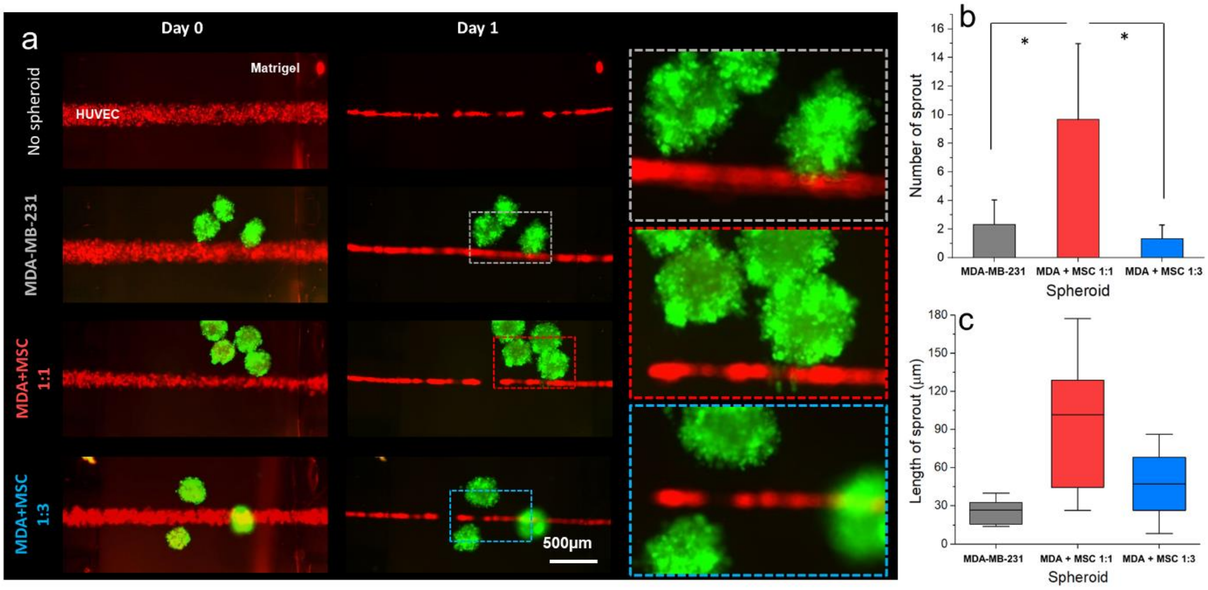

Figure S1. Invasion of tumor spheroid mixtures in Matrigel. (a) Tumor spheroids-HUVEC vasculature interaction in a microfluidic chip device. Enlarged images of each highlighted spheroid area are given on the right side of (a). The number of sprouts (n=3, respectively) (b) and the length of sprouts (c) in each spheroid culture condition were quantified. Green cells represent GFP-expressing MDA-MB-231; red cells represent mApple-expressing HUVECs.

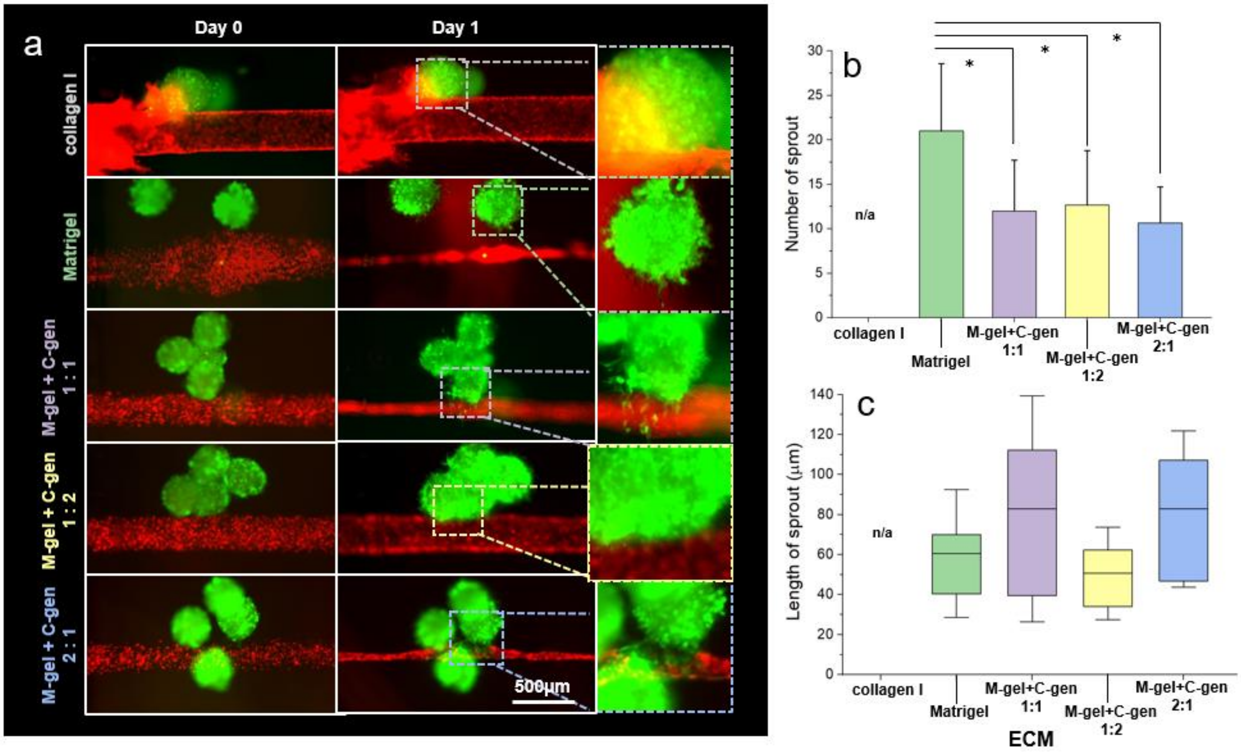

Figure S2. Spheroids of MDA-MB-231 and MSCs in Matrigel and collagen I matrices. (a) The 1:1 mixture of MDA-MB-231 metastatic breast cancer cells and MSCs tumor spheroids were introduced into the microfluidic chip device embedded in different ECM microenvironments. Enlarged images of each highlighted spheroid area are given the right side. The number (b) and length of sprouts (c) in each spheroid were quantified under each condition (n=3, respectively). Green cells represent GFP-expressing MDA-MB-231; red cells represent mApple-expressing HUVECs.

Tumor spheroids in groups 2-5 formed invasive sprouts into the bioengineered HUVEC vessel, the number and length of which are plotted in **Figure S2b and c**. The average number of sprouts from spheroids cultured in the 1:2 mixture of Matrigel and collagen I (group 4) was 12.67 ± 6.13. This was slightly more than that of those cultured in the 1:1 and 2:1 mixture of Matrigel and collagen I ECM (groups 3 and 5), which produced on average 12 ± 5.72 and 10.67 ± 4.03 sprouts, respectively (**Table S1 and Figure S2b**). However, the sprouts produced in the 1:2 mixture of Matrigel and collagen I had an average length of 24.75 ± 11.14 μm, which is substantially shorter

than those produced in the 1:1 and 2:1 mixture of Matrigel and collagen I (40.48 ± 27.23 μm and 82.81 ± 38.44 μm, respectively) (**Table S2 and Figure S2c**).

Table S1. Number of sprouts

ECM	Collagen I	Matrigel	M-gel+C-gen 1:1	M-gel+C-gen 1:2	M-gel+C-gen 2:1
Number of sprouts	n/a	21 ± 7.58	12 ± 5.72	12.67 ± 6.13	10.67 ± 4.03

Table S2. Length of sprouts

ECM	Collagen I	Matrigel	M-gel+C-gen 1:1	M-gel+C-gen 1:2	M-gel+C-gen 2:1
Number of sprouts	n/a	29.63 ± 15.52	40.48 ± 27.23	24.75 ± 11.14	40.46 ± 18.78
